# OVATE family gene *CmOFP6-19b* negatively regulates fruit size in melon (*Cucumis melo* L.)

**DOI:** 10.1093/hr/uhaf148

**Published:** 2025-06-18

**Authors:** Junling Chi, Haimei Yan, Wenjing Zhang, Dingfang Tian, Gen Che, Agula Hasi

**Affiliations:** Key Laboratory of Herbage & Endemic Crop Biology, Ministry of Education, School of Life Science, Inner Mongolia University, 235 Daxue West Street, Saihan District, Hohhot 010070, China; Key Laboratory of Herbage & Endemic Crop Biology, Ministry of Education, School of Life Science, Inner Mongolia University, 235 Daxue West Street, Saihan District, Hohhot 010070, China; Key Laboratory of Herbage & Endemic Crop Biology, Ministry of Education, School of Life Science, Inner Mongolia University, 235 Daxue West Street, Saihan District, Hohhot 010070, China; Key Laboratory of Herbage & Endemic Crop Biology, Ministry of Education, School of Life Science, Inner Mongolia University, 235 Daxue West Street, Saihan District, Hohhot 010070, China; Key Laboratory of Herbage & Endemic Crop Biology, Ministry of Education, School of Life Science, Inner Mongolia University, 235 Daxue West Street, Saihan District, Hohhot 010070, China; Key Laboratory of Herbage & Endemic Crop Biology, Ministry of Education, School of Life Science, Inner Mongolia University, 235 Daxue West Street, Saihan District, Hohhot 010070, China

## Abstract

OVATE family proteins (OFPs) constitute a class of transcription factors regulating various developmental processes in plants. Nevertheless, their precise regulatory functions in melon (*Cucumis melo* L.) fruit development remain elusive. In this study, we identified expression profiling of melon *OFP* genes and revealed the molecular function of *CmOFP6-19b* gene mediating fruit size variation. Quantitative analysis revealed predominant *CmOFP* expression in reproductive organs (female/male flowers and ovaries), with distinct differential expression patterns observed among paralogs. Through melon genetic transformation, we revealed that *CmOFP6-19b* gene functions as a negative regulator in fruit enlargement. Overexpression of the *CmOFP6-19b* gene resulted in reduced fruit size, while its downregulation led to increased fruit size. Bimolecular fluorescence complementation and yeast two-hybrid assays confirmed nuclear-localized physical interaction between CmOFP6-19b and CmKNOX16. Overexpression of *CmKNOX16* in melon produced smaller fruits, phenocopying the *CmOFP6-19b*-Oe lines. Quantitative real-time PCR (RT-qPCR) analysis showed negative correlation between *CmOFP6-19b*/*CmKNOX16* expression level and fruit size, with peak expression levels observed in a cultivar displaying minimal longitudinal diameter. The results of histological section and expression analysis suggest that *CmOFP6-19b* and *CmKNOX16* may affect melon fruit size by regulating genes related to cell division and cell expansion. In conclusion, our findings systematically characterized the phylogenetic architecture and expression divergence of *CmOFP* genes, and elucidated the function and molecular mechanism of CmOFP6-19b-CmKNOX16 regulatory module in mediating melon fruit development, providing a theoretical foundation for melon breeding.

## Introduction

Melon (*Cucumis melo*), which belongs to the Cucurbitaceae crops, is characterized by its sweet flavor and rich nutritional content, with significant economic value and global cultivation [1, 2]. In melon agronomic trait research, fruit size is a crucial factor affecting yield and quality. Generally, fruit size is quantitatively assessed by measuring the longitudinal and transverse diameters of the fruit. The genetic regulation underlying melon fruit size determination involves a complex network of 26 *FS* (Fruit Size) consensus QTLs (quantitative trait loci), which were identified in melon [3–5]. Among them, only *CmOFP1a* (*CmFSI8/CmOFP13*), *CmACS7*, and *CmCLV3* were validated as the candidate genes for the *FS* locus [5, 6]. *CmACS7* is not only involved in flower sex determination, but also contributes to the elongated morphology of the fruit by coordinating cell division and cell expansion [7, 8]. *CmCLV3* was able to regulate the variation of melon carpel number and then affect fruit shape [9, 10]. Overexpression of *CmCRC* and *CmSUN23-24* led to melon fruit elongation [11, 12]. The *OVATE* gene was colocalized with melon fruit morphology QTL and found to regulate fruit shape and size [13–15]. Despite QTL analyses and several gene characterization being conducted in melon, the genetic regulation mechanism of the key regulators remains largely unknown.

In model horticultural plant tomato, *OVATE* gene was identified as a pivotal regulator in fruit development [16]. OVATE family proteins (OFPs) constitute a unique class of plant-specific transcription factors that extensively participate in diverse plant growth and developmental processes [17]. *OVATE* was first isolated in tomato (*Solanum lycopersicum*), where a single nucleotide polymorphism led to a morphological transition in fruit shape from spherical to pyriform [16]. Currently, phylogenetic analyses revealed OFP orthologs in many plant species, including model plant (*Arabidopsis*, tomato) and commercial crops (cucumber, grape, mango, strawberry, and wax melon) [18–24]. Strong overexpression of *SlOFP20* interrupted the normal pollination process and led to sterility, while mild overexpression of *SlOFP20* reduced the fruit length and increased fruit width, producing flattened and smaller fruits [13, 25]. Ectopic overexpression of grape *VvOFP4* gene in both tomato and tobacco led to alterations in cellular shape, subsequently influencing the morphogenesis of both nutrient and reproductive organs [21]. Ectopic expression of cucumber *CsOFP12-16c* gene in *Arabidopsis* reduced silique length with apical blunting. Conversely, the knockdown of *CsOVATE* resulted in longer fruit neck, suggesting that *OFP* genes negatively regulate cucumber longitudinal growth [20, 26]. Regulation of *OFP* gene expression alters plant organ shape and size, and different *OFP* genes may be functionally redundant within this process. In melon, *CmOFP13*, underlying *CmFSI8* locus, controls fruit shape development. Ectopic expression of *CmFSI8/CmOFP13* in *Arabidopsis* resulted in kidney-shaped leaves and shortened siliques [6]. In rice, *OsOFP19* forms functional protein complexes with OSH1 and DLT1, regulating brassinosteroid-mediated growth regulation and signaling homeostasis [27]. In *Arabidopsis*, AtOFP5 interacts with KNAT3 and BLH1 to regulate embryo sac development [28]. While conserved functions of OFP family members have been characterized in model crops/plants, the specific regulatory networks governing melon fruit development await systematic investigation.

**Figure 1:**
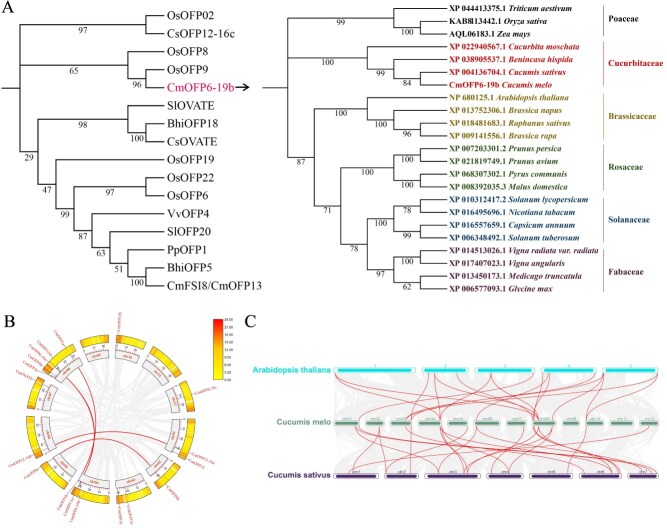
Phylogenetic analysis and collinear analysis of *OFPs*. (A) Phylogenetic tree was reconstructed using CmOFP6-19b and functionally characterized OFP orthologs implicated in fruit morphogenesis across angiosperms. CmOFP6-19b clustered within a conserved cucurbit-specific clade. (B) Chromosomal mapping revealed the segmental duplication events among CmOFPs. In the inner circle, melon chromosomes are scaled to physical distances. The outer circle indicates the density of genes on the chromosome. Solid lines indicate segmental duplication of *CmOFP* gene pairs. (C) Genome collinear relationship of OFP paralogs in melon, cucumber, and *Arabidopsis*. Solid lines indicate the collinear *OFP* gene pairs between different genomes.

Here, we performed comprehensive analysis of sequence characteristic and expression profiling of the melon *CmOFPs.* Meanwhile, we found the function of *CmOFP6-19b* in melon fruit development and explored the negative role of CmOFP6-19b-CmKNOX16 module in regulating fruit size.

**Figure 2:**
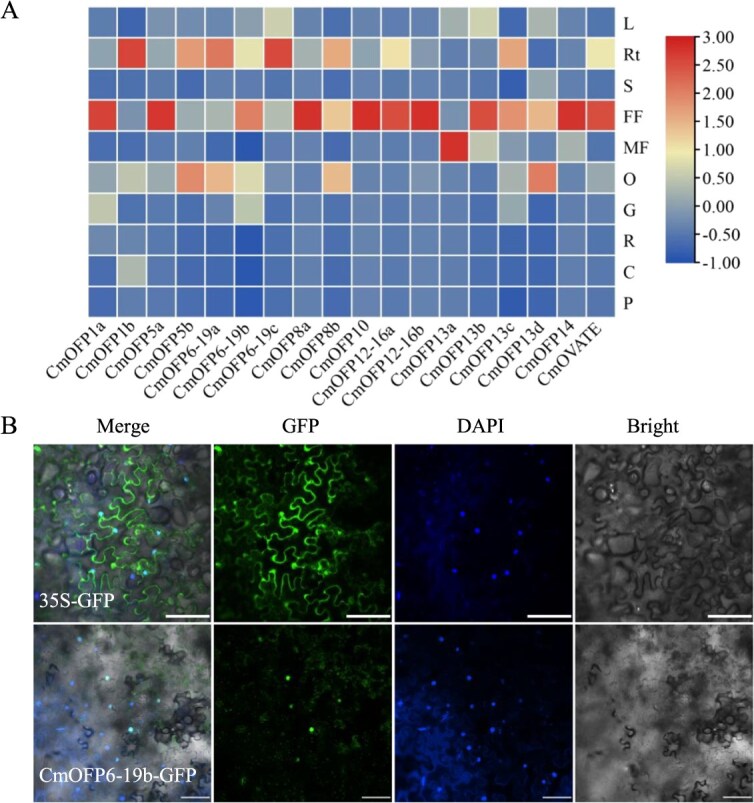
Expression profile of *CmOFP* genes in different tissues and subcellular localization of CmOFP6-19b in *N. benthamiana* leaves. (A) The heatmap was generated using TBtools based on RT-qPCR results. L, Rt, S, FF, MF, O, G, R, C, and P represent leaf, root, stem, female flower, male flower, ovary, fruit at growing stage, fruit at ripening stage, fruit at climacteric stage, and fruit at postclimacteric stage, respectively. Three biological replicates and three technical replicates were performed for each gene. (B) Subcellular localization of CmOFP6-19b was performed in *N. benthamiana* leaves. Empty vector transformation was used as negative control. Bar = 100 μm.

## Results

### Structural and evolutionary analysis of CmOFPs

Genome-wide characterization identified 18 OVATE-domain containing proteins (CmOFPs) in melon [5]. Notably, *CmOFP6-19b* (MELO3C009515) exhibits phylogenetic affinity with established fruit morphogenesis regulators (*OsOFP8*, *OsOFP9*), strongly suggesting conserved regulatory functions in fruit architecture determination (Fig. 1A). Comparative analysis showed that *OFP* genes in Cucurbitaceae were clustered in one phylogenetic clade (Fig. 1A). The molecular characteristics of *CmOFPs* such as gene ID, chromosome location, CDS and protein length (AA), molecular weight (MW), isoelectric point (PI), and predicted subcellular localization are listed in Table S1. Five evolutionarily conserved motifs (motif 1–5) in CmOFP proteins were identified in melon (Fig. S1). All CmOFPs contain OVATE domain, motif 1, and motif 2, indicating critical structural roles in maintaining OVATE domain architecture. Gene structure analysis demonstrated an intron-less architecture in most (17/18) *CmOFP* genes, with *CmOFP8b* being the sole exception containing two exons (Fig. S1), reflecting strong evolutionary conservation of exon–intron organization. Gene duplication is one of the primary processes in genetic evolution. Collinearity analysis identified four segmental duplication events involving 10 *CmOFP* paralogs (*CmOFP1a*, *CmOFP1b*, *CmOFP8a*, *CmOFP8b*, *CmOFP6-19a*, *CmOFP6-19b*, *CmOFP13a*, *CmOFP13d*, *CmOFP12-16a*, and *CmOFP12-16b*) (Fig. 1B), suggesting tandem duplication as a major driver of *CmOFP* family expansion. Cross-species synteny analysis revealed 23 orthologous gene pairs between *C. melo* and *Cucumis sativus OFPs*, versus 11 pairs with *Arabidopsis thaliana* (Fig. 1C), indicating stronger evolutionary conservation within Cucurbitaceae (Fig. 1C). To decipher transcriptional regulation mechanisms, we analyzed 2-kb promoter regions upstream of *CmOFP* genes, identifying 12 functional cis-regulatory elements. The hormone-responsive elements (ABRE, CGTCA-motif, GARE-motif, TCA-element, and AuxRR-core) and light-responsive elements (Box4, G-Box, and GT1-motif) were identified in the *CmOFPs* promoter region. Statistical analysis showed ABRE (ABA), CGTCA-motif (JA), and GARE-motif (GA) occurred in 61%, 55%, and 44% of *CmOFP* promoters, respectively, indicating strong hormonal regulation potential (Fig. S2).

**Figure 3:**
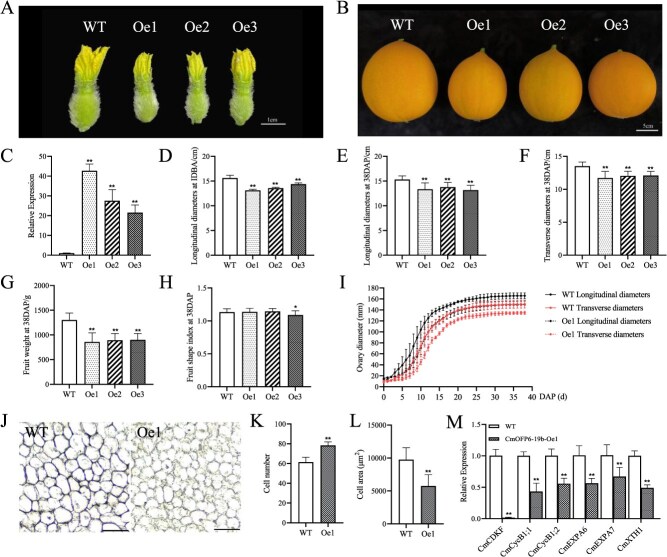
Overexpression of *CmOFP6-19b* inhibits melon fruit size. (A) Ovary phenotype of WT and *CmOFP6-19b*-Oe at the DBA. Bar = 1 cm. (B) Mature fruit phenotype of WT and *CmOFP6-19b*-Oe lines. Bar = 5 cm. (C) Expression level of *CmOFP6-19b* in WT and Oe lines. (D) Longitudinal diameter of ovary at 1 DBA in WT and *CmOFP6-19b*-Oe lines (*n* = 20). (E–H) Longitudinal diameter, transverse diameter, weight and fruit shape index of mature fruits at 38 DAP in WT, and Oe lines (*n* = 20). (I) Growth curves of WT and *CmOFP6-19b*-Oe1 fruits. (J) Longitudinal sections of WT and *CmOFP6-19b*-Oe1 fruits at 10 DAP. Bar = 200 μm. (K, L) Measurement of cell number and cell area displayed in Panel J. (M) Expression of cell division- and cell expansion-related genes in melon fruit. ^**^P < 0.01 and ^*^P < 0.05, Student’s *t*-test.

### Expression profile of *CmOFPs* and subcellular localization of CmOFP6-19b

To study the transcription levels of *CmOFP* genes in melon, we used RT-qPCR to examine the relative expression of all 18 *CmOFPs* in 10 developmental tissues (root, stem, leaf, male flower, female flower, ovary, and fruit at four different stages) (Fig. 2A). The results revealed that most of *CmOFP* genes were highly expressed in the female flowers, while a few of them exhibited specific expression in roots, ovaries, or male flowers. Notably, almost all *CmOFP* genes showed low expression in fruits. The expression of *CmOFP1a*, *CmOFP5a*, *CmOFP8a*, *CmOFP10*, *CmOFP12*-*16a*, *CmOFP12*-*16b*, *CmOFP13b*, *CmOFP14*, and *CmOVATE* was higher in female flower, whereas *CmOFP13a* was highly expressed in male flower. During the fruit development, only four genes, *CmOFP1a*, *CmOFP1b*, *CmOFP6-19b*, and *CmOFP13c*, showed transcript accumulation. Among them, *CmOFP6-19b* displayed broad expression patterns with peak accumulation in female flower, followed by ovary, root, female flower, and G-stage fruits, suggesting pleiotropic regulatory functions during plant development (Fig. 2A). The above results suggested that *CmOFP* genes mainly participate in the development of melon reproductive organs. Based on its unique spatiotemporal expression profile, *CmOFP6-19b* was prioritized for functional characterization. *CmOFP6-19b* coding sequence without the termination codon was fused to the *GFP* fragment driven by the 35S promoter. We transformed 35S::*CmOFP6-19b*:*GFP* construct into *Nicotiana benthamiana* leaves via *Agrobacterium* infiltration and detected the fluorescence by confocal microscope. The nuclear enrichment of CmOFP6-19b-GFP overlapping DAPI-stained nuclei was detected (Fig. 2B), confirming that CmOFP6-19b was localized to the plant nucleus.

### Overexpression of *CmOFP6-19b* significantly reduced fruit size in melon

To determine the function of *CmOFP6-19b* in melon, we overexpressed *CmOFP6-19b* gene in melon by genetic transformation. Three independent overexpression transgenic lines showed significantly elevated expression level of *CmOFP6-19b* gene and altered fruit morphology (Fig. 3A–C). Reduction of ovary size was observed in *CmOFP6-19b*-Oe lines, with longitudinal diameter reduced by 15.8% (Oe1: 13.1 ± 0.2 mm vs WT 15.6 ± 0.6 mm; *P* < 0.01) at 1 day before anthesis (DBA) (Fig. 3D). Quantitative analysis of mature fruits (38 DAP) also revealed consistent reduction in fruit size and fruit weight. The Oe lines had significantly reduced fruit longitudinal diameter, transverse diameter, and weight by 9.93%–13.39%, 10.54%–13.28%, and 30.85%–34.24%, compared with WT mature fruits (Fig. 3E–G), while fruit shape index (FSI = longitudinal/transverse diameter) remained unaltered in Oe1 and Oe2 lines compared to WT, except for Oe3 line (Fig. 3H). Next, we followed the fruit morphology of WT and *CmOFP6-19b*-Oe1 fruits from pollination to the ripening stage. The growth phase progression of WT and *CmOFP6-19b*-Oe1 fruits was generally similar (Fig. 3I). Both longitudinal and transverse diameters of both fruits showed a faster growth rate from 1 to 20 days after pollination (DAP), and the growth rate gradually leveled from 20 to 32 days and stabilized after 32 days. Interestingly, the longitudinal and transverse diameters of *CmOFP6-19b*-Oe1 fruits were always smaller than those of WT fruits throughout the growth cycle (Fig. 3I). The longitudinal sections of WT and *CmOFP6-19b*-Oe1 fruits at 10 DAP showed that the transgenic fruits had an increased cell number and decreased cell area compared to WT (Fig. 3J–L). Through expression analysis of cell division- and cell expansion-related genes, we found that the expression levels of cell cycle regulatory genes (*CDKF*, *CycB1;1*, and *CycB1;2*) and cell expansion-related genes (*EXPA* and *XTH*) were significantly suppressed in the transgenic plants compared to WT (Fig. 3M). These findings establish *CmOFP6-19b* as a negative regulator of melon fruit size, operating through cell division and expansion processes.

### Knockdown of *CmOFP6-19b* led to increased melon fruit size

**Figure 4:**
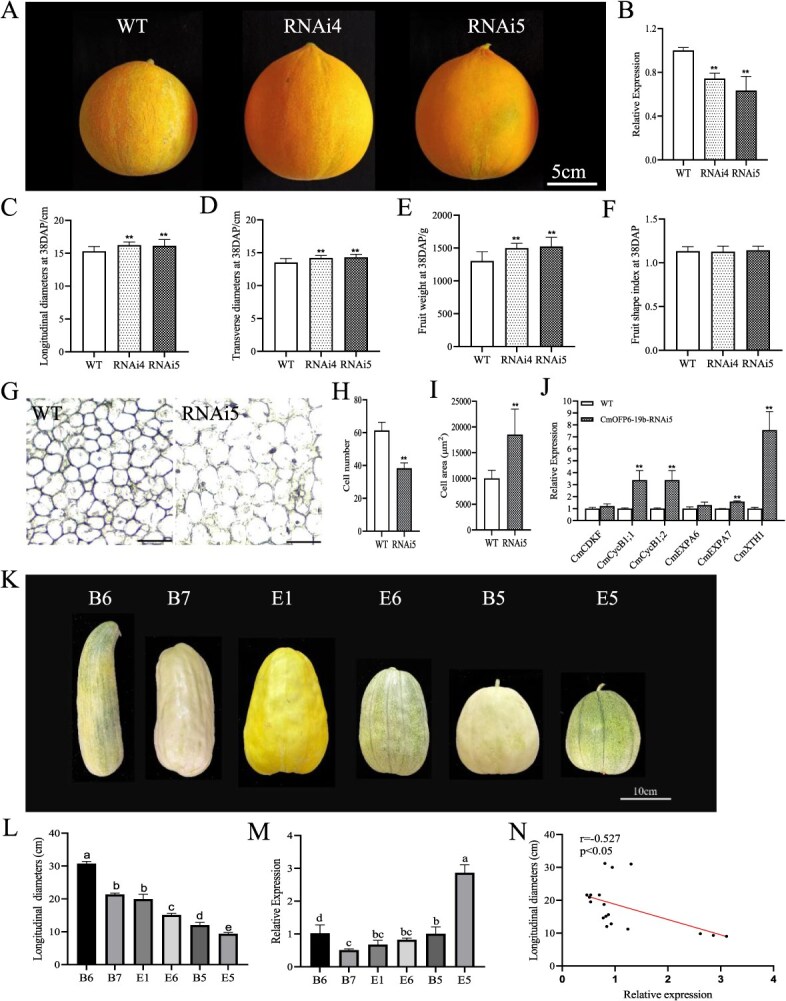
Downregulation of *CmOFP6-19b* resulted in reduced melon fruit size. Expression level of *CmOFP6-19b* in different melon cultivars. (A) Mature fruit phenotype of WT and *CmOFP6-19b-*RNAi lines. Bar = 5 cm. (B) Expression level of *CmOFP6-19b* in WT and RNAi lines (^**^P < 0.01, Student’s *t*-test). (C–F) Mature fruit longitudinal diameter, transverse diameter, weight and fruit shape index of WT, and RNAi lines, respectively (*n* = 20, ^**^P < 0.01, Student’s *t*-test). (G) Longitudinal sections of WT and *CmOFP6-19b*-RNAi5 fruits at 10 DAP. Bar = 200 μm. (H, I) Cell number and cell area (*n* = 10) displayed in Panel G (^**^P < 0.01, Student’s *t*-test). (J) Expression of cell division- and cell expansion-related genes in *CmOFP6-19b*-RNAi5 fruit. (K) Fruit shape observation of different melon cultivars, B6, B7, E1, E6, B5, and E5. Bar = 10 cm. (L) Statistical analysis of fruit longitudinal diameter in different melon cultivars. (M) Expression level of *CmOFP6-19b* in different melon cultivars. Significance analysis was performed using one-way ANOVA. (N) Pearson correlation analysis showed negative correlation between the *CmOFP6-19b* expression level and the melon fruit longitudinal diameter.

We introduced *CmOFP6-19b* into melon under 35S promoter and identified two independent RNAi lines by assessing the *CmOFP6-19b* gene expression level in plants (Fig. 4A and B). Phenotypic characterization revealed that *CmOFP6-19b*-RNAi lines produced significantly enlarged fruits (Fig. 4A and B). In particular, the longitudinal diameter and transverse diameter of RNAi lines were increased by 6.11%–6.69% and 5.10%–6.65% with respect to WT (Fig. 4C and D). Fruit weight analysis showed 14.9% ± 16.9% increase in RNAi lines (RNAi5: 1523.8 ± 139.0 g vs WT 1302.5 ± 141.8 g, *P* < 0.01) (Fig. 4E). There was no difference in FSI between the *CmOFP6-19b*-RNAi lines and WT fruits (Fig. 4F). Longitudinal sections of WT and *CmOFP6-19b*-RNAi5 fruits showed a reduction in cell number and an increase in cell area compared to WT (Fig. 4G–I). Transcriptional profiling revealed significant upregulation of cell cycle progression genes *CmCycB1;1* and *CmCycB1;2*, along with cell wall remodeling genes *CmEXPA7* and *CmXTH1* in RNAi5 fruits, and unaffected expression of *CmCDKF* and *CmEXPA6* (Fig. 4J). Natural variation analysis across six melon accessions revealed significant intercultivar divergence in longitudinal diameter (30.7–9.4 cm), with cultivar E5 displaying minimal dimension (9.4 ± 0.4 cm) (Fig. 4K and L). RT-qPCR quantification identified 2.86-fold higher *CmOFP6-19b* expression in E5 fruits compared to B6, inversely correlating with longitudinal diameter (*r* = −0.527, *P* < 0.05) (Fig. 4M). This result suggested that there is a negative correlation between the expression level of *CmOFP6-19b* gene and the longitudinal diameter in melons (Fig. 4N).

### CmOFP6-19b and CmKNOX16 have a direct protein interaction

**Figure 5:**
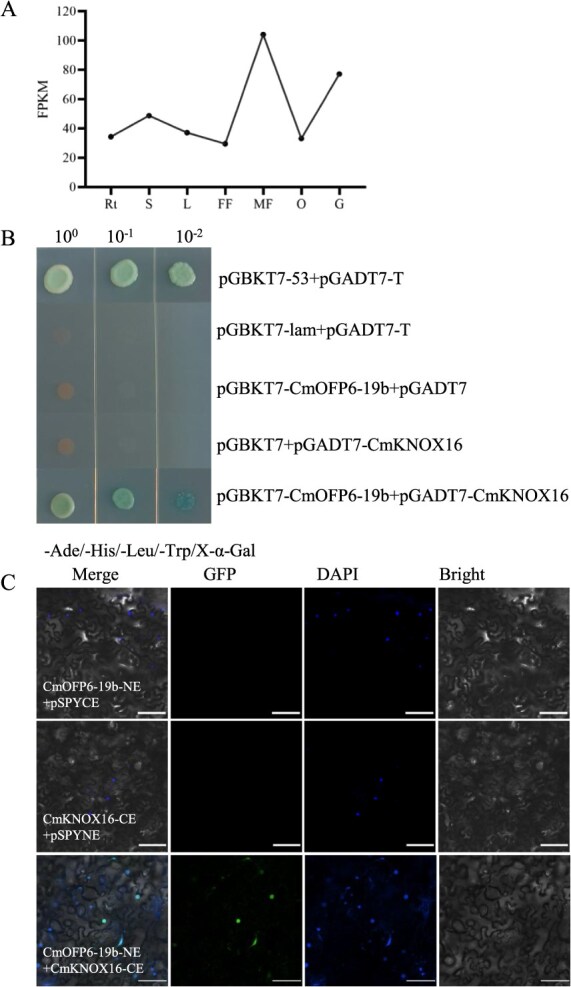
Expression pattern of *CmKNOX16* and interaction assays of CmOFP6-19b and CmKNOX16. (A) The expression pattern of *CmKNOX16* in different melon tissues. Rt, S, L, FF, MF, O, and G represent root, stem, leaf, female flower, male flower, ovary, and growing fruit. (B) Yeast two-hybrid assay. Positive interaction was examined by SD/−Ade/−His/−Leu/−Trp/−X-α-gal. pGBKT7-53 and pGADT7-T were used as positive controls, pGBKT7-lam and pGADT7-T were used as negative controls. (C) Bimolecular fluorescence complementation assay. *CmOFP6-19b*-NE and *CmKNOX16-*CE were coexpressed in *N. benthamiana* leaves. Empty vectors coexpressed with corresponding recombinant vectors were used as negative controls. Bar = 100 μm.

Evolutionary conservation analysis revealed OFP-KNOX functional interaction in different species, with characterized interactions in *Arabidopsis*, rice, and cotton. In melon, we identified a total of 21 CmKNOX proteins through genome-wide identification and named CmKNOX1–CmKNOX21 with chromosomal distribution (Fig. S3, Table S2). STRING database prediction identified CmKNOX16 as the candidate interactor of CmOFP6-19b, validated by the spatiotemporal coexpression pattern in male flower and growing fruit (Fig. 5A). Yeast two-hybrid assays confirmed that CmOFP6-19b had no transcriptional autoactivation and CmOFP6-19b interacted with CmKNOX16 (Fig. 5B). In addition, the interaction was further verified by bimolecular fluorescence complementation in *N. benthamiana* leaves. CmOFP6-19b and CmKNOX16 coexpression in the tobacco epidermal cells exhibited strong fluorescence of nuclear colocalization (Fig. 5C).

### Overexpression of *CmKNOX16* inhibits melon fruit size

**Figure 6:**
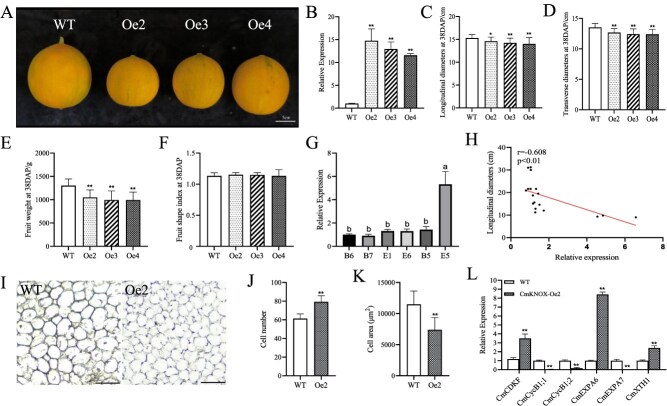
Overexpression of *CmKNOX16* inhibits melon fruit size. Expression level of *CmKNOX16* in different melon cultivars. (A) Mature fruit phenotype of WT and *CmKNOX16-*Oe lines. Bar = 5 cm. (B) Expression level of *CmKNOX16* in WT and Oe lines (^**^P < 0.01, Student’s t-test). (C–F) Mature fruit longitudinal diameter, transverse diameter, weight and fruit shape index of WT, and Oe lines, respectively. (*n* = 20, ^**^P < 0.01 and ^*^P < 0.05, Student’s *t*-test). (G) Expression level of *CmKNOX16* in different melon cultivars. Significance analysis was performed using one-way ANOVA. (H) Pearson correlation analysis showed negative correlation between longitudinal diameter and *CmKNOX16* expression level. (I) Longitudinal sections of WT and *CmKNOX16*-Oe2 fruits at 10 DAP. Bar = 200 μm. (J, K) Cell number and cell area (*n* = 10) displayed in Panel I (^**^P < 0.01, Student’s *t*-test). (L) Expression of cell division- and cell expansion-related genes in *CmKNOX16* Oe2 fruit (^**^P < 0.01, Student’s *t*-test).

To characterize the gene function of *CmKNOX16* in melon fruit development, *CmKNOX16* overexpression vector was transformed into melon. Three independent *CmKNOX16*-Oe lines showed significantly smaller fruits compared to WT fruit (Fig. 6A and B). Compared with WT fruits, *CmKNOX16*-Oe fruit showed reduced fruit longitudinal/transverse diameter, decreased fruit weight, and invariant FSI (Fig. 6C–F). The expression level of *CmKNOX16* gene in different melon cultivars showed peak expression in E5 with the smallest longitudinal diameter (Fig. 6G). Correlation analysis revealed that the expression level of the *CmKNOX16* gene was negatively correlated with the melon fruit longitudinal diameter (Fig. 6H). In comparison with WT, the longitudinal sections of WT and *CmKNOX16*-Oe2 fruits had increased cell number and enlarged cell area (Fig. 4I–K), with altered expression of several cell division and cell expansion genes (Fig. 4L). The results indicated that *CmKNOX16* negatively regulates fruit size in melon, which is similar to the involvement of *CmOFP6-19b* in melon fruit regulation. This functional congruence between *CmKNOX16* and *CmOFP6-19b* suggests their cooperative action in a conserved regulatory module.

## Discussion

The *OVATE* gene family, encoding plant-specific transcription factors, plays a pivotal role in modulating plant growth and development. In melon, fruit morphology constitutes critical agronomic traits of commercial importance. While previous investigations have identified numerous quantitative trait loci (QTL) and candidate genes associated with melon fruit morphology [5, 29], the molecular mechanisms underlying these genetic determinants remain largely unexplored. Notably, the OVATE family proteins (OFPs), particularly those implicated in fruit shape regulation, have been systematically characterized and functionally validated across multiple plant species, demonstrating their essential roles in governing fruit morphogenesis [20, 21, 24, 30]. Our structural analysis revealed that most *CmOFP* genes exhibit intron-less architectures, with the exception of *CmOFP8b* (Fig. S1), a genomic organization pattern consistent with *OFP* homologs in cucumber [20]. This structural feature may confer evolutionary advantages, as intron-deficient genes are postulated to enhance environmental adaptability through rapid transcriptional responses [31, 32]. Spatial expression profiling demonstrated predominant *CmOFP* transcript accumulation in floral organs and developing ovaries (Fig. 2), mirroring expression patterns observed in cucumber orthologs [19, 20]. This conserved expression signature implies evolutionary maintenance of OFP-mediated regulatory mechanisms in cucurbit fruit development.

Melon fruit morphology is predominantly determined during pre-anthesis developmental stages through precise regulation of ovary development [33–35], a phenomenon corroborated in related cucurbits, including watermelon and cucumber [36, 37]. The temporal–spatial expression pattern of *CmOFP6-19b*, characterized by high transcript levels in female flowers, ovaries, and development fruits, strongly suggests its regulatory involvement in melon morphogenesis. Quantitative phenotypic analysis revealed significant reductions in longitudinal diameter, transverse diameter, and fruit weight in *CmOFP6-19b*-Oe transgenic lines compared to WT (Fig. 3). Conversely, the fruits of transgenic lines with *CmOFP6-19b*-RNAi showed an opposite trend (Fig. 4), indicating this gene as a negative regulator of fruit size development. This functional conservation extends to *SlOFP20* in tomato, which similarly inhibits fruit expansion [13, 25]. Moreover, subsequent analysis showed that *CmOFP6-19b* gene expression was negatively correlated with the fruit longitudinal diameter in melon cultivars (Fig. 4), reinforcing its inhibitory regulatory role. Fruit development is fundamentally governed by the coordinated regulation of cell proliferation and expansion processes [38, 39]. Cell cycle regulatory gene (*CDK*) and *Cycs* genes play important roles in cell division [40], as well as *Expansions* (*EXPA*) [41, 42] and *XTH*, which mediate cell wall loosening and directional organ elongation [43, 44]. In the *CmOFP6-19b*-Oe1 fruit, the expression levels of both cell division- and cell expansion-related genes, were significantly repressed (Fig. 3). In contrast, the expression of these genes was upregulated in *CmOFP6-19b*-RNAi5 fruit (Fig. 4). Notably, these genes were also observed to be differentially expressed in *CmKNOX16* overexpression fruit (Fig. 6). The findings suggest that *CmOFP6-19b* and *CmKNOX16* may affect fruit size by regulating the cell division and cell expansion processes.

**Figure 7:**
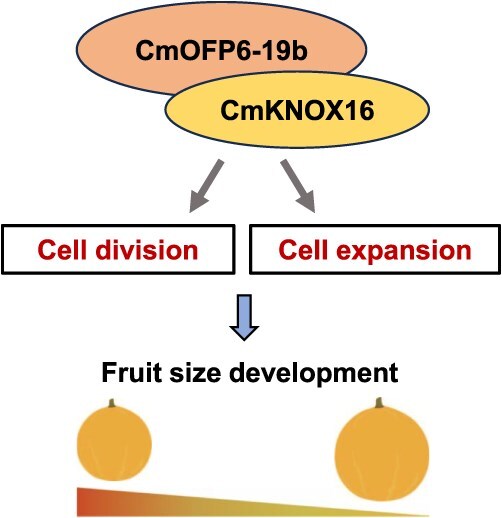
A proposed model for the regulatory mechanism of *CmOFP6-19b* in controlling fruit size.

Many functional OFP proteins frequently mediate developmental processes through interactions with homeodomain transcription factors, particularly KNOX/BELL heterodimers. *OsOFP2* coordinates seed shape and lignin synthesis through interaction with the BLH-KNOX complex in rice [45]. In *Arabidopsis*, *AtOFP4* regulates secondary cell wall formation through interaction with KNAT7 [46]. Our protein interaction assays confirmed physical association between CmOFP6-19b and CmKNOX16 (Fig. 5), suggesting their cooperative regulation of melon fruit development. The interactions between OFP and KNOX proteins have been reported in various model plants including *Arabidopsis* [28, 46], tomato [25], and rice [27, 45], exhibiting remarkable functional plasticity across angiosperm lineages, as evidenced by its species-specific regulatory specialization. In *Arabidopsis*, these protein complexes orchestrate secondary wall biosynthesis through GA pathway modulation [28, 46], whereas OsOFP19-OSH1 in rice coordinates leaf morphogenesis via brassinosteroid crosstalk [27]. Our findings reveal that in melon, the CmOFP6-19b-CmKNOX16 complex converges on conserved cell division and cell expansion targets (Figs 3–6). It is similar to suppression of SlOFP20-KNOX1 in tomato fruit development [25], suggesting fleshy fruits may have conserved cis-regulatory adaptations in evolution. *KNOX* transcription factors are involved in plant growth and development by regulating the activity of meristematic tissues [47]. In tomato, *KNOX* gene *TKA-II* constrained fruit size variation through gibberellin-mediated processes [48]. In rice, *KNOX* gene *HOS59* resulted in inhibited kernels of development [49]. In melon, phenotypic characterization of *CmKNOX16-*Oe lines revealed parallel reductions in fruit dimensions and weight, accompanied by similar negative correlations with longitudinal expansion (Fig. 6). These congruent functional profiles of *CmOFP6-19b* and *CmKNOX16* implicate their synergistic action in melon fruit size regulation, potentially through modulation of cell expansion/division-related gene networks (Fig. 7). Notably, our findings establish *CmOFP6-19b* and *CmKNOX16* as pleiotropic developmental modulators coordinating melon fruit morphogenesis, providing crucial molecular targets for marker-assisted selection strategies in cucurbit breeding programs. The phylogenetically conserved OFP-KNOX interaction module, now substantiated in melon through this investigation, constitutes an evolutionary innovation fundamental to plant organogenesis and environmental adaptation. Nevertheless, the key mechanistic aspects in their epistatic relationships within hormone signaling pathways and potential post-translational modifications influencing complex formation need to be clarified in further investigation.

## Conclusion

The study provides comprehensive bioinformatic characteristics of the *CmOFPs* gene family and functional validation of *CmOFP6-19b* as a negative regulator of melon fruit size. The demonstrated interaction between CmOFP6-19b and CmKNOX16 establishes a regulatory module, potentially operating through transcriptional control of cellular division or expansion mechanisms. These findings not only elucidate molecular determinants of melon fruit morphology but also offer valuable genetic targets for precise breeding of commercial melon cultivars.

## Materials and methods

### Plant materials and growth conditions

Hetao melon (*C. melo* cv. Hetao) inbred lines were cultivated under standard agriculture condition in Dengkou county, Inner Mongolia region. Six representative melon cultivars, E1, B7, B5, B6, E6, and E5, were selected for developmental analysis, with mature fruits harvested at physiological ripening stage (R-stage). Significant variations in longitudinal diameter were observed among cultivars (detailed measurements provided in Table S3). Hetao melon seedlings were cultivated in an artificial climate chamber under conditions of 25°C with a 16-h/18°C light period and an 8-h/18°C dark period at 60% relative humidity. Various plant tissues, including roots, stems, young leaves, ovaries at the anthesis day, female flowers, male flowers, and fruit at growing stage (growing [G], ripening [R], climacteric [C], and postclimacteric [P] stages), were collected. All samples were frozen in liquid nitrogen and stored at −80°C.

### Gene structure and promoter analysis

Gene structure, conserved motifs, and domains analysis of *CmOFP* genes were performed using TBtools software. Then, 2000-bp upstream sequences of *CmOFP* genes were extracted from melon genome database, and cis-elements were analyzed using PlantCARE (https://bioinformatics.psb.ugent.be/webtools/plantcare/html/).

### Phylogenetic tree construction and collinearity analysis

The phylogenetic tree was performed using MEGA11 software. The protein sequences of CmOFP6-19b homologs and functionally characterized OFPs in other plants were downloaded from NCBI (https://blast.ncbi.nlm.nih.gov/Blast) and listed in Table S4. We downloaded the genome files and GFF annotation files of melon, cucumber, and *Arabidopsis* from CuGenDB (http://cucurbitgenomics.org) and TAIR (http://www.arabidopsis.org/), respectively. MCScanX was used to identify tandem duplication events of *CmOFP* genes within the melon species and to analyze the syntenic relationship of homologs in *Arabidopsis* and cucumber.

### Melon transformation and RT-qPCR

Full-length coding sequences of *CmOFP6*-*19b* and *CmKNOX16* were cloned into the pBI1305 vector. A 495-bp DNA fragment of *CmOFP6-19b* was amplified, and both sense and antisense fragments were inserted into the pFGC1008 vector. To obtain transgenic plants, recombinant plasmids of overexpression vectors and RNAi construct were introduced into melon female flower by the ovary injection method [50–52]. The recombinant plasmids (pBI1305-*CmOFP6-19b*, pBI1305-*CmKNOX16*, pFGC1008-*CmOFP6-19b*) were suspended in 0.1× SSC (pH 7.0) and its concentration was adjusted to 100 ng/μl concentration and then stored at −20°C. 6–7 hours after the self-pollination, the petals of the female flowers were removed to expose the stigma. Ten-microliter diluted recombinant plasmid delivery was achieved using sterile microinjectors, with strict adherence to bubble-free aspiration. The injection needle penetrated 2/3 of the ovarian depth along the pollen tube pathway, followed by gradual solution administration. Twenty biological replicates per construct resulted in 50%–60% fruit set efficiency with injected specimens. The transformed seeds were collected from mature fruits and germinated under controlled conditions. At the five-leaf developmental stage, genomic DNA was extracted from seedling leaves for PCR-based transgene detection. Positive transgenic seedlings were transferred to a greenhouse for phenotypic evaluation, followed by artificial self-pollination to obtain homozygous progeny. Pulp tissues from mature fruits (38 DAP) of both transgenic lines and WT plants were used for RT-qPCR analysis of target gene expression levels. Seeds of the identified *CmOFP6-19b* and *CmKNOX16* transgenic melon lines were propagated and cultivated under controlled conditions, during which morphological characteristics and phenotypic were recorded.

To explore the transcript levels of *CmOFP* genes in different melon tissues, we reverse-transcribed the extracted total RNA into cDNA. The synthesized cDNA used as a template for RT-qPCR analysis, with *CmGAPDH* used as internal reference gene. The results of RT-qPCR were calculated by 2^–ΔΔCT^ method and visualized through heatmaps using TBtools. All RT-qPCR experiments for detecting gene expression levels (Figs 2–4 and 6) were performed with three biological and three technical replicates. The specific primers used for RT-qPCR used were listed in Table S5.

### Subcellular localization

The coding sequences of *CmOFP6-19b* excluding the stop codon were cloned into pCAMBIA1300-GFP expression vector. The primers used are listed in Table S6. pCAMBIA1300-*CmOFP6-19b-*GFP recombinant vector was transformed into *Agrobacterium* GV3101 and then injected into the lower epidermis of 4-week-old leaves of *N. benthamiana*. The infiltrated plants were subsequently maintained at ambient temperature (25°C) for 60 hours. Following infiltration, leaf samples were harvested and stained with DAPI solution at a final concentration of 0.5 μg/ml, followed by incubation in darkness in 5 minutes. The leaf samples were rinsed twice with 0.9% saline solution to eliminate unbound DAPI dye, and then the leaves were prepared for microscope observation. Fluorescence signal was observed using a laser scanning confocal microscope with an excitation wavelength of 488 nm.

### Yeast two-hybrid assay

Full-length coding sequences of *CmOFP6-19b* and *CmKNOX16* were inserted into pGBKT7 and pGADT7 vectors, respectively. The primers were listed in Table S6. The following combinations, pGBKT7-*CmOFP6-19b* + pGADT7, pGADT7-*CmKNOX16* + pGBKT7, and pGBKT7-*CmOFP6-19b* + pGADT7-*CmKNOX16*, were cotransformed into the yeast strain AH109. Single-yeast strains grown on the SD/−Trp/−Leu selection plates were individually transferred into PCR tubes with 10 μl of ddH_2_O for resuspension and subsequent dilution. Yeast suspensions were prepared at three different concentrations: 10^0^, 10^-1^, and 10^–2^, respectively. Yeast suspensions at different concentrations were spotted onto the SD/−His/−Leu/−Trp solid selection medium supplemented with 4 mg/ml X-α-gal for the both activation detection and protein–protein interaction analysis. pGBKT7-53 + pGADT7-T was used as a positive control and pGBKT7-lam + pGADT7-T as a negative control.

### Bimolecular fluorescence complementation assay

Full-length sequences of *CmOFP6-19b* and *CmKNOX16* excluding the termination codon were inserted into pSPYNE-35S and pSPYCE-35S, respectively. The primers were listed in Table S6. The combinations of *CmOFP6-19b*-NE + pSPYCE, *CmKNOX16*-CE + pSPYNE, and *CmOFP6-19b*-NE + *CmKNOX16-CE* were cotransformed into *Agrobacterium* GV3101 and injected into the abaxial leaf epidermis of *N. benthamiana* for approximately 60 hours. The infected leaves were initially stained with 0.5 μg/ml DAPI solution, then rinsed with 0.9% saline solution before microscope observation. Fluorescence signals were detected using a laser confocal microscope with 488-nm excitation wavelength.

### Paraffin sectioning

Transgenic melon fruits (*CmOFP6-19b-*Oe1, *CmOFP6-19b-*RNAi5, and *CmKNOX16-*Oe2) and WT controls were harvested at 10 DAP. Fresh pulp tissues from the equatorial region were immediately fixed in 3.7% formaldehyde-acetic acid solution under vacuum infiltration for 24 hours at 4°C. Following gradient ethanol dehydration (50%, 70%, 85%, 95%, and 100%) samples were embedded in paraffin wax. The samples were sectioned and observed under a fluorescence microscope. Cell number and cell area were measured using Image J software. Pairwise comparisons between transgenic and WT lines were performed using GraphPad Prism 9, with significance thresholds set at *P* < 0.05 (*).

## Supplementary Material

Web_Material_uhaf148

## Data Availability

All experimental data are available in the main text and supplementary data. RNA-Seq data in this study can be found at the NCBI SRA (https://www.ncbi.nlm.nih.gov/sra); the BioProject accession numbers are PRJNA543288 and PRJNA803327.
