# Supplementary material for: OVATE family gene CmOFP6-19b negatively regulates fruit size in melon (Cucumis melo L.)
Source: Hortic Res. 2025 Jun 18;12(9):uhaf148. doi: 10.1093/hr/uhaf148 (PMC12373640; doi:10.1093/hr/uhaf148)
Supplement: Web_Material_uhaf148 [file web_material_uhaf148.zip › Supplementary Figure revised.docx]

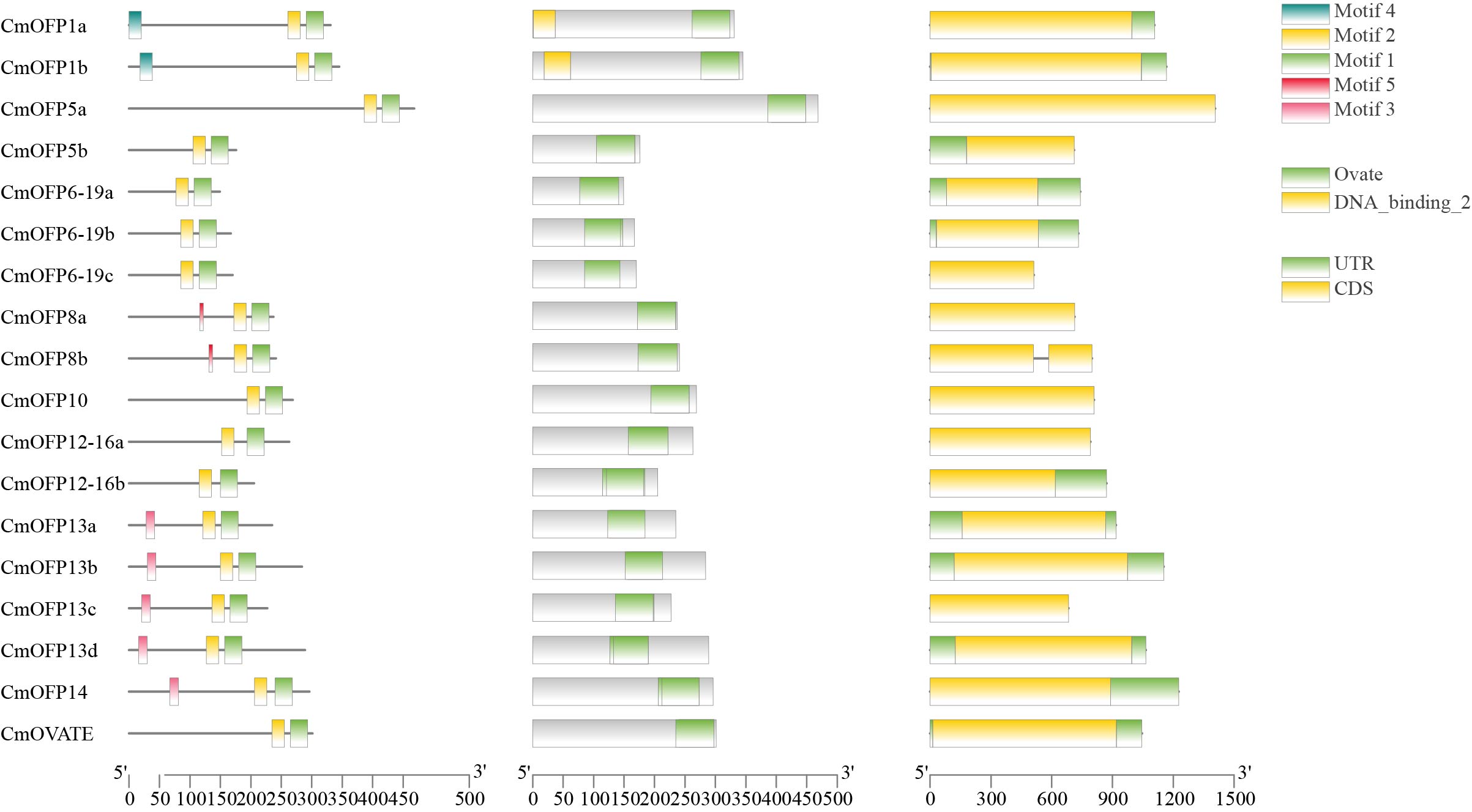


Fig. S1 Conserved motif, conserved domain and gene structure of CmOFPs. Five motifs were identified and different color boxes indicate different motif. Yellow boxes and green boxes indicate the locations and sizes of different domains. Yellow boxes and green boxes indicate exon and UTR, respectively. Black line indicate intron.


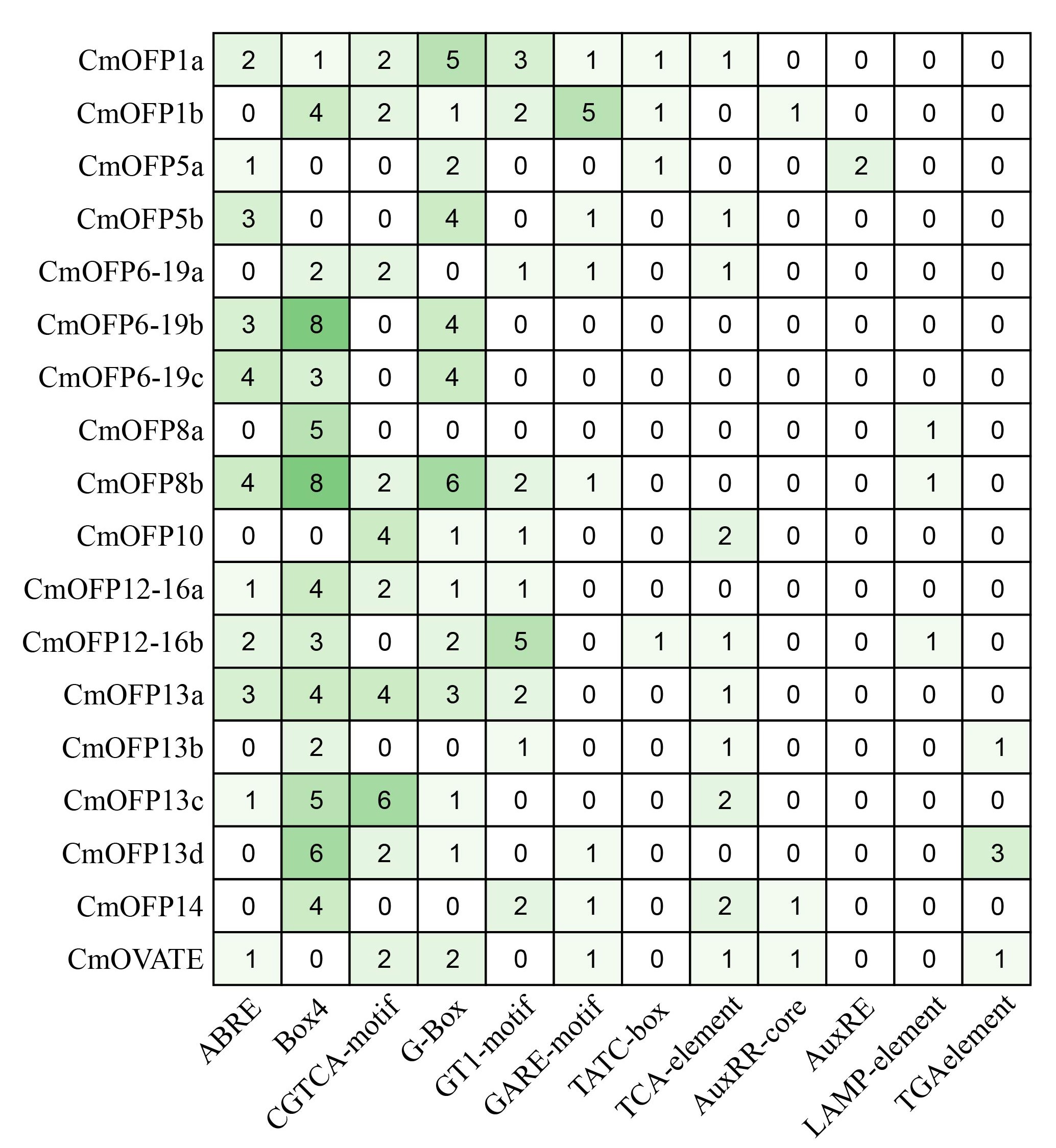


Fig. S2 Number of each cis-element of *CmOFP* genes promoter region in melon. The number represents the number of cis-acting elements.


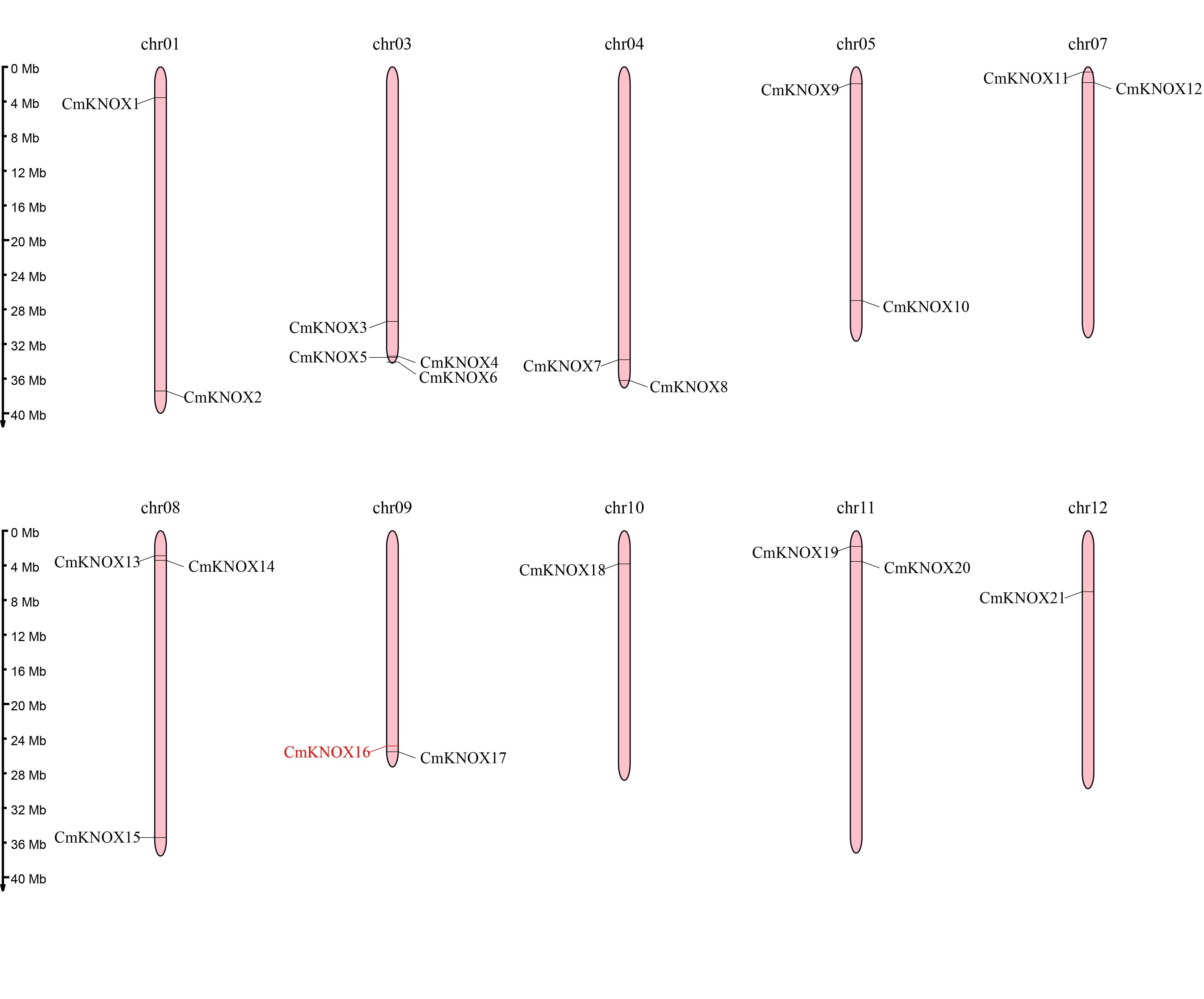


Fig. S3 Chromosomal localization of the *KNOX* genes family in melon
