# Supplementary material for: OVATE family gene CmOFP6-19b negatively regulates fruit size in melon (Cucumis melo L.)
Source: Hortic Res. 2025 Jun 18;12(9):uhaf148. doi: 10.1093/hr/uhaf148 (PMC12373640; doi:10.1093/hr/uhaf148)
Supplement: Web_Material_uhaf148 [file web_material_uhaf148.zip › Supplementary Table revised.docx]

Table S1 *CmOFPs* detail information

| Gene ID | Rename | Chromosome  location | CDS (bp) | Protein length (aa) | PI/MW (KD) | Subcellular  localization |
| --- | --- | --- | --- | --- | --- | --- |
| MELO3C025206.2 | CmOFP1a | Chr8 | 996 | 331 | 9.81/37.2 | Nucleus |
| MELO3C009113.2 | CmOFP1b | Chr4 | 1038 | 345 | 9.83/38.7 | Nucleus |
| MELO3C007193.2 | CmOFP5a | Chr8 | 1407 | 468 | 9.59/54.4 | Nucleus |
| MELO3C007422.2 | CmOFP5b | Chr8 | 531 | 176 | 7.58/20.4 | Nucleus |
| MELO3C024574.2 | CmOFP6-19a | Chr8 | 450 | 149 | 7.91/16.9 | Nucleus |
| MELO3C009515.2 | CmOFP6-19b | Chr4 | 504 | 167 | 9.54/18.6 | Golgi apparatus/ Nucleus |
| MELO3C025581.2 | CmOFP6-19c | Chr12 | 513 | 170 | 6.95/19.2 | Nucleus |
| MELO3C004557.2 | CmOFP8a | Chr5 | 714 | 237 | 10.03/27.5 | Nucleus |
| MELO3C025343.2 | CmOFP8b | Chr2 | 726 | 241 | 9.42/27.6 | Nucleus |
| MELO3C010932.2 | CmOFP10 | Chr3 | 810 | 269 | 9.6/30.9 | Nucleus |
| MELO3C015818.2 | CmOFP12-16a | Chr1 | 792 | 263 | 5.08/29.0 | Nucleus |
| MELO3C006531.2 | CmOFP12-16b | Chr6 | 618 | 205 | 6.04/23.0 | Nucleus |
| MELO3C009514.2 | CmOFP13a | Chr4 | 708 | 235 | 5.75/25.9 | Nucleus |
| MELO3C012340.2 | CmOFP13b | Chr10 | 855 | 284 | 5.08/31.9 | Nucleus |
| MELO3C019910.2 | CmOFP13c | Chr3 | 684 | 227 | 5.08/25.9 | Chloroplast/Cytoplasm/Nucleus |
| MELO3C024573.2 | CmOFP13d | Chr8 | 870 | 289 | 4.39/32.1 | Nucleus |
| MELO3C024232.2 | CmOFP14 | Chr1 | 798 | 265 | 8.18/29.9 | Nucleus |
| MELO3C017554.2 | CmOVATE | Chr7 | 906 | 301 | 9.28/34.5 | Nucleus |

Table S2 Gene ID and chromosome location of *CmKNOX* genes

| Gene ID | Rename | Chromosome Location |
| --- | --- | --- |
| MELO3C018844.2.1 | CmKNOX1 | chr01 |
| MELO3C023409.2.1 | CmKNOX2 | chr01 |
| MELO3C011281.2.1 | CmKNOX3 | chr03 |
| MELO3C010775.2.1 | CmKNOX4 | chr03 |
| MELO3C030120.2.1 | CmKNOX5 | chr03 |
| MELO3C010678.2.1 | CmKNOX6 | chr03 |
| MELO3C009542.2.1 | CmKNOX7 | chr04 |
| MELO3C009220.2.1 | CmKNOX8 | chr04 |
| MELO3C014519.2.1 | CmKNOX9 | chr05 |
| MELO3C004181.2.1 | CmKNOX10 | chr05 |
| MELO3C016988.2.1 | CmKNOX11 | chr07 |
| MELO3C016850.2.1 | CmKNOX12 | chr07 |
| MELO3C007417.2.1 | CmKNOX13 | chr08 |
| MELO3C007500.2.1 | CmKNOX14 | chr08 |
| MELO3C003175.2.1 | CmKNOX15 | chr08 |
| MELO3C005620.2.1 | CmKNOX16 | chr09 |
| MELO3C005699.2.1 | CmKNOX17 | chr09 |
| MELO3C011979.2.1 | CmKNOX18 | chr10 |
| MELO3C023333.2.1 | CmKNOX19 | chr11 |
| MELO3C020878.2.1 | CmKNOX20 | chr11 |
| MELO3C004927.2.1 | CmKNOX21 | chr12 |

Table S3 Melon cultivars information.

| Melon | Average longitudinal diameter | Average transverse diameter | Average fruit weight |
| --- | --- | --- | --- |
| E1 | 19.9±1.5 cm | 12.5±0.2 cm | 1793.2±316.9 g |
| E5 | 9.4±0.4 cm | 9.5±0.7 cm | 506±62.9 g |
| E6 | 15.1±0.6 cm | 12.3±1.1 cm | 859.3±116.3 g |
| B5 | 12.0±0.8 cm | 9.4±0.5 cm | 1259.2±96.4 g |
| B6 | 30.7±0.6 cm | 6.9±0.6 cm | 868.7±23.2 g |
| B7 | 21.4±0.4 cm | 9.4±0.9 cm | 1275.7±169.8 g |

Table S4 The protein sequences of CmOFP6-19b and other OFPs in different plants

| Name | Protein sequence |
| --- | --- |
| CmOFP6-19b | MAAVKKKLLINTISVDIGCGSCRKSKSIISQIFRPKPKSPTSYSDRRLFRSLSSSSEKKLSDSDMAYAPEVVGGGGFWKIGGVSVAVEKDSNDPYVDFRQSMLQMILENEIYTQEGLRELLSCFLHLNSPCNHGIIIRAFAEIWDGVFCARSAAPAKQRRHVRSRAF |
| CmFSI8/CmOFP13 | MRNHKFRFSDMIPNAWFYKLKEIGGASRPKSFRSNKNPHHPPPPPPPSKHKQPPPPPPHSRSRKSYYFTRQLESNDAYFVNSPPPSPPLLPVPIPPRKSTKQLKPGRKQTSSRSSAKLLSSSSVGCSCHTTAESIWTKSDSPPEFSTSPSDTSPDFRTDKILTAEASKHFEHDIVIDVSSNYSNNAVIGAFDELELPPIITKQRKKTETKQRTTTTTTAGTKKVAGNSPGVRLRIHSPKIGYRKMGGRKSVSSRRSLSESLAIMKSSYDPQKDFRESMVEMIVENNIRGSKELEDLLACYLCLNADEYHDLIIKVFKQIWFDLTQPSPPPL |
| CsOVATE | MMMTPKRFKLLRIPSFHCCRSNDISVVPTDPPSPPPPPKPHHSSLRRHVSSAFRTAACGCRSSSTNSDDDQICKSSPTLPTHVPPTPLLHSFDDGSTFPKRQRRRKNKKKSKSKSTTLTRLRTSTSSTESGLFSSESFDEIDELEETETLISSSKTISTSDDDNDSSSEFNPQLETIREKPNKINLRRKKEKEKRRRKQKRTTIISPSPEIESPARLSVFQRLIPCTVEGKIRESFAVVKKSADPFEDFKRSMMEMIMEKEMFEEKDLEQLLHCLLSLNDREHHGIIVEAFSEIWQSLFCN |
| CsOFP12-16c | MSNLKFLNNLYSFFSKLKFSPPPPVIASHTPPSDCYFTSNPISSTTADDCDDFFSTSSDADDSISDDLAALLASRRFFFSSPGRSNSIFEYSSCSRRQQPHDVLVSEGHRIRKYSMDPYADFRRSMQEMVEARELEDVRSDSEFLRELLSCYLRLNPKNTHKFIVKAFSDLVLSLLASSSPTPAPASIARRKVVTSR |
| SlOVATE | MGKSLKLRFSRVIASFNSCRSKNPSSLPQNPNFFPHKLTSTKHISPDFPLIDQNQNQNHRNYVPESTMISVGCCRSEFKWEKEEKFHVVSSSFVSEEEECEEEINLALRPPLTPPRFSRIVVEKKKKKQQRVKKTKTKSRIIRMSTSSADEYSGVLSGTNTDWDNNEEETETLVSSSR |
| SlOFP20 | MGNYRFRLSDMMPNAWFYKLKDMAKSSSRRHSHTTSSSNLQLDKKRQPHNNLGCQRKSYYISRNLTITSPISSNSPKLDHNVHITEPSRKSYKKRRSTNFRRRNSPKPVNSSASVESVWTKPDSTPEQYPNSSSSSSSSSPSSILPHKSNPIASISPSCDCRTDYTNQNSANLDPGVHSVSKIDLPRIITKPEKFNEKIQEKQRIVKQEQRIVRRVSTNGVKLRTNSPRITTTTTNSRKSVSSKRTSVTTDSFAVVKSSRNPQKDFRESMVEMIIENNITTSKDLEELLACYLSLNSDEYHDIIIKVFKQIWFEITEIRLK |
| BhiOFP5 | MVEMIVENNIRGSKELEDLLACYLCLNADEYHDLIINVFKQIWFDLTQPPL |
| BhiOFP18 | MMMAKRFKLLRIPSFHCCRSNDISVVPTDPPSSLPPPKPHRSSLRRHVSSAFKTATCGCRSSSTNSDDDQISKSSPTFPTHVPPTPLLDPYDDVFALPKRRRRRRKKKFKSTTLAQLRISTSSAESGLFSSESFDENDQLEETETLISSSKTMSSTDDDNDSSSQFNPQLETIREKPYKTKNKNNLRTNKEKEKRHRSSNRRQKQNRTIIKTSPSPEIESPARLSVFQRLIPCTVEGKIRESFAVVKKSADPFEDFKRSMMEMIMEKEMFDEKDLEQLLHCLLSLNDREHHGIIVEAFSEIWQSLFCN |
| OsOFP2 | MQEDNISKKAKLRKSIQLFLSRNLKKIPPIHIPSSAIPAKITSNRLLSTCRFPRTPSLHGGGGGGGDHRTTDDDDGSNGRDQAATLSDVDRFLFDNFRSLYIHDGDNHQQRRQPPPSPGKFTQPAAAETSSSRSESVAEDARGTSSGDEDDNNSSTAIMLFSVDPYTDFRRSMQNIIEMHHGEEPQPLDWDFLEELLFYYLQLNEQSVHKYILKAFADLTAGAHVSCPARGKPQWADKSVRSRKHY |
| OsOFP6 | MGRHKFRLSDMIPNAWFFKLRDMRAARGGAGAGGGGASHGGVVTQSSVAVSRAGRACRPLPNTPRHGALSLPHRASYYYTPRAGDLLVGSPLHPKCSDTQFPPLQLSPPRKSRRRHRRRSVKLAPSVSGSSVLSSPVSTGCRCGRKPELVVVEAPDTPPCRRDKFVGYNDDDDDEEEEEVEFKKPTVAVAACDELDGKVITSATDIIIDLRTEKRPDKVLPPIVTKPARRELDGCDLEEKHIDVVRRASAKKPTTLLEQSKPRRSVSSARRLKTRANTPRIVAKKSKPPPPPPPAAARSPAPTTKPPLAESFAVVKSSRDPRRDFRESMEEMIAENGIRTAADLEDLLACYLSLNAAEYHDLIVDVFEHIWANLADIKM* |
| OsOFP8 | MSGRSSRRGSFSLRQPPVVDIGCNCRRPKLFSIFSSSSSSSFRRGGSKPKSPNASSTSTTTAFTATTGGAGTATSTDSSWGPASFTTNSLFEEPAAAQQEQEQLETRRRRRQQRRRRRRAGATSFARGGDVGGHDDEQQQLQEQAPYRRVAKESVAVAVESAEPYEDFRESMVQMVVEKEIYAWDDLNDLLHQFLSLNSPRHHPLILHAFADLWTRNGLFSPPSPCQF |
| OsOFP9 | MMSPGVSAKKRHAGAGFTLGCGCKDAKSVSVSASAAGTPSTTATRRRSAGTNPSGSTTTDTLTMTSASSSFLWEHSVVEFDHDGGGGCGPESFSGLLRELSELEQSVASWGRKSHHQHHDKKHSPAPSSPLPPQEDRKEKNGGNGDATDKPGDCRDGGGGGDGVGVALDGSVAVVKQSDDPLGDFRQSMLQMIVENGIVAGEDLREMLRRFLTLNAPHHHDVILRAFAEIWDGVFAATASLVHHHHPPPSSRREPVAPAARPPAPRTPPRHRHPSPRAWRV |
| OsOFP19 | MSSHERFRLSHLMPNSWFYKLRDMKRPRPTSSRMITAADHAARSSRRSSSSSSSIHHYYLHGHGTTTPKPLPLSPPRRSYYPYLERAKQMPLMEKESQLISHSPLHQRIPATAIPGDHHDGEFQDLQLRPIRTRPPSAAAASAEPRRTASGSGTCPSSPRMRSRRLHVLGGCECRAGSGRRRSGGGGFAVVKASAEPARDFRESMVEMVVGNGMRSPEDLLELLECYLSLNAREHHGVIMEAFRGVWVEIVADADCCVGL |
| OSOFP22 | MLSSEPGVLTGRYGPPFSETRKEQKEQRNLAPHALSRRYLSRAGHGVVALWPGPPFDLSLLLHSSSSYSAEERRRRRRRAGERRAREDMGRRKFRLSDMMPNAWFYKLRDMRARGGRGATAMQPPSSSSLMRGSRAAQQQAGTWRLGTSSSSSSLLPHRASYYYTTRDREVPPLPPPPPPRGVDDQFPSLTLSPPLPTRNSRRRHRVGRFGSTEMDGGELVLAPSDDHDGCSHQEPPVADASGSSRCRRDMFIGRDGGRGVEFRRRATTVDGPEEDAAVDVKVITSDADIIIDLGADDDDDTPERVLRPVVTRPARRELDWCEPAEVKHVDLAELMTPRASSASASSEKSISTGKPRRSSVSSRRRLKTRTNSPRLAACRKGKPTARATTTTPTQPPLAHSFAVVKTSSDPRRDFLESMEEMIAENGIRDAGDLEDLLACYLSLNSGEYHDLIVEVFEQVWTGLAAACGVMP |
| VvOFP4 | MGNYRFRLSDMMPNAWFYKLKDMSRARNHNTAQSIKKKLPSPAATSQKTNLSQPRYSYYYNEEPIRADMLYGSPVNPKASDTHFPDPPRKSSKRRPKRKTIYKPSPTLVSSVSAGYGCPAAFESVWTEHDPTESEDCFVSAIEPGHNVSLLPEVGPNTDVASDTFNGLAPLSTSCSCRVCSSTTDIFIDVSKKSFSSKVEKLDEFDTVSELELPQVSTKPARFRETTVEAARHRRSSSQLEEIKAQRSLSIKIVKEGSKQTQKEPKANPLVRKPTANAAGVKLRTNSPRLASRKIQAYARKSVYSRTSSKAQSRSLSESCAVVKASFDPERDFRDSMLEMIVENNIRASKDLEELLACYLSLNSDEYHDLIVKAFEQIWFDTSDLRM |
| PpOFP1 | MGNHKFRLSDMMPNAWFHKLKDMSKPRKNPNSPHPSKKKKQQQKPTFASTAKFTEPSKPKQQLPHQCLPRQSYYFTRELTSAAPGHRFCSSSPTNPKASDTNFPDPPPKKPSKQKPKKRITSLPSDPHLVTSSVSAGCGCRAPIESVWTKSDSPPELWSSSTLDSSPEPESHDEDDGELELHEPEFRCDRVLATETFDGMVSMSSSCAAYLADSEEKDVVIDVDKASLSMKLSDVKLSDMADNGLYSFSELELAPIITKPPKFSEMVRDVKKKKETKEPSRCRRSSAKFQDRNAHGSLSVKVAKEESTSTKTIKEQRTASSVRRVSSNATSPGVRLRMNSPRIANRKINQANLSRRSVSSNSSSKRRSLSESFAIVKSSFDPQRDFRESMVEMIMENNIKASKDLEDLLACYLSLNSDEYHELIIKVFKQIWFDLTDLRSK |

Table S5 The primer sequence for RT-qPCR

| Gene | Forward sequence | Reverse sequence |
| --- | --- | --- |
| CmOFP1a | ACAACGGCGGAATCTATCTGGAC | GATTACGATGTCGTGCTCGAAGTGT |
| CmOFP1b | ACATGACGACCATTATTAGGCGAAG | AGAATTGGCAGCAAGTTGGGAG |
| CmOFP5a | AGTGAATGGGAATCGTAATCGTCT | TCCTCTGAGTTGTACCACACTGACC |
| CmOFP5b | GAGCCTTGTGTTGTGGATGTTCTT | TGAGCGATGCTGGAAACTGATG |
| CmOFP6-19a | ATCTCCAGCCCACTTCCCTCT | GGATTTGAGGGAGGGCTTTG |
| CmOFP6-19b | GCTCCTCATCAACACCATCTCCG | CGACGAGCTGGATAGTGAACGG |
| CmOFP6-19c | CTCTGACATCGTCCAACCTAACAAG | CGTCGAGAGAGAAGGTGCCAT |
| CmOFP8a | CGCCTATTCCAATCCTCATTCTTC | GGGAGTTTGGGTGTGTAATTAGAGG |
| CmOFP8b | TACAGTAGCGAGGATGAAGACGACG | CTATTTCTGTCGTCGGTGAGATGCT |
| CmOFP10 | CATCTCCTCTTTCCGATTCTGCC | GGTGAGGGTGGATTGGAGTAACAG |
| CmOFP12-16a | ACCCTCTCTCCGCCACTCAAT | GTGGTGATCTCGTAGAGGGAGTTGT |
| CmOFP12-16b | CCCTGGCTGCTCCAACTCTATT | AAACCTTCCGAACTTCTACGCC |
| CmOFP13a | GCAGCCTTTCCACGGTATCG | CACGGTCTCGTCTTCCACTATCG |
| CmOFP13b | CCAACCCTTCGAAACCATCACT | AGTTGGTAGCAGAGACAGAATCGG |
| CmOFP13c | AATCCGCTAGGGTTTCGCTC | TCTGATTTCCCGCCTCTGG |
| CmOFP13d | CATCTTTCGAACCATCAACTCTGC | AAGGCCTCAATTTGGTCTTCATC |
| CmOFP14 | GGTTGTAAGCATCCAAAGACCCTAT | AAAGAGAAATCGGTCGATATCGG |
| CmOVATE | AACTCCAACTCTCCCGACGC | AACTCCAACTCTCCCGACGC |
| CmCDKF | GATTTGTGGTCATTGGGCTGTAT | CAGAAGAACAGTTGGTAAGACATGC |
| CmCycB1;1 | GCCTCAGGAAGCAAGCCAAAT | GGCAAGCGGGTTATCGGAAT |
| CmCycB1;2 | GGTCCCTGTTGTGCCAATACTGT | TTATTGGTCTTTTCGGGCTTCTC |
| CmEXPA6 | GAACTCAGAATCAGCCCACCATAT | TGTTGTTATTCCCAGTTGAAGTACC |
| CmEXPA7 | GCTTGTGGGTATGGGAATTTGT | GAAATTAGTGGCGGTGACGAT |
| CmXTH1 | CAAGGAAAGGGAAATAGGGAACAAC | TGGGGGTTCCAAATGATAGAGTAAG |
| CmGAPDH | GTTTTCACCGACAAAGACAAAGCTG | TCCGGCTTGTATTCCTTCTCGTTAAC |

Table S6 The primers for vector construction

| Primer | Primer sequence (5’-3’) | vector |
| --- | --- | --- |
| OFP6-19b-Oe-F | GC**TCTAGA**ATCATGGCCGCCGTAAAG | pBI1305 |
| OFP6-19b-Oe-R | CG**GGATCC**AGGCGACGGTTAGAAGGCG |  |
| OFP6-19b-AscI-F1 | TT**GGCGCGCC**ATCATGGCCGCCGTAAAG | pFGC1008 |
| OFP6-19b-SwaI-R1 | G**ATTTAAAT**GAAGGCGCGTGACCGTAC |  |
| OFP6-19b-SpeI-F2 | GG**ACTAGT**ATCATGGCCGCCGTAAAG |  |
| OFP6-19b-BamHI-R2 | CG**GGATCC**GAAGGCGCGTGACCGTAC |  |
| OFP6-19b-SL-F | CG**GGATCC** ATCATGGCCGCCGTAAAG | pCAMBIA1300 |
| OFP6-19b-SL-R | GC**TCTAGA**GGCGCGTGACCGTACATGGCGTCT |  |
| OFP6-19b-Y2H-F | CC**CATATG**ATCATGGCCGCCGTAAAG | pGBKT7 |
| OFP6-19b-Y2H-R | CG**GGATCC**TTAGAAGGCGCGTGACCGT |  |
| OFP6-19b-BiFC-F | CG**GGATCC**ATCATGGCCGCCGTAAAG | pSPYNE |
| OFP6-19b-BiFC-R | GG**GGTACC**GGCGCGTGACCGTACATGGCGTCT |  |
| KNOX16-Oe-F | GC**TCTAGA**ATGCAAGAACCTGGGTTGGGAAT | pBI1305 |
| KNOX16-Oe-R | CG**GGATCC**TTATCGCTTGCGCTTGGACTTC |  |
| KNOX16-Y2H-F | CC**CATATG**ATGCAAGAACCTGGGTTGGGAAT | pGADT7 |
| KNOX16-Y2H-R | CG**GGATCC**TTATCGCTTGCGCTTGGACTTC |  |
| KNOX16-BiFC-F | GC**TCTAGA**ATGCAAGAACCTGGGTTGGGAAT | pSPYCE |
| KNOX16-BiFC-R | CG**GGATCC**TCGCTTGCGCTTGGACTTC |  |
